# Evidence of Diel Vertical Migration in *Mnemiopsis leidyi*

**DOI:** 10.1371/journal.pone.0086595

**Published:** 2014-01-22

**Authors:** Matilda Haraldsson, Ulf Båmstedt, Peter Tiselius, Josefin Titelman, Dag L. Aksnes

**Affiliations:** 1 Department of Biological and Environmental Sciences, University of Gothenburg, Fiskebäckskil, Sweden; 2 Department of Ecology and Environmental Sciences, Umeå University, Umeå, Sweden; 3 Department of Biosciences, University of Oslo, Oslo, Norway; 4 Department of Biology, University of Bergen, Bergen, Norway; University of Wales Swansea, United Kingdom

## Abstract

The vertical distribution and migration of plankton organisms may have a large impact on their horizontal dispersal and distribution, and consequently on trophic interactions. In this study we used video-net profiling to describe the fine scale vertical distribution of *Mnemiopsis leidyi* in the Kattegat and Baltic Proper. Potential diel vertical migration was also investigated by frequent filming during a 24-hour cycle at two contrasting locations with respect to salinity stratification. The video profiles revealed a pronounced diel vertical migration at one of the locations. However, only the small and medium size classes migrated, on average 0.85 m h^−1^, corresponding to a total migration distance of 10 m during 12 h. Larger individuals (with well developed lobes, approx. >27 mm) stay on average in the same depth interval at all times. Biophysical data suggest that migrating individuals likely responded to light, and avoided irradiance levels higher than approx. 10 *µ*mol quanta m^−2^ s^−1^. We suggest that strong stratification caused by low surface salinity seemed to prohibit vertical migration.

## Introduction

The vertical distribution and migrations of planktonic organisms may affect their large scale spatial distribution and dispersal, e.g. review in [1]. Perhaps the most pronounced and well described migration pattern among zooplankton is the diel vertical migration (DVM), where organisms leave the productive surface layers and migrate deeper during day. To reduce predation pressure from visual predators by abandoning the surface layers during daylight is commonly agreed to be the major ultimate reason for DVM [2]. Light and light changes have often been characterized as the proximate cue for DVM [3], [4], but also other factors such as sight or smell (i.e. kairomones) of predators, food concentration, and temperature [3], [5] may enhance or inhibit DVM. For higher trophic levels however, such as top-predators, migrating behavior may sometimes be explained by the tracking of a migrating prey [2], [6].

DVM is common among the true jellyfishes [7]–[10], and both light mediated [11] and zooplankton tracking [6] have been suggested. For the ctenophore phyla however, DVM has rarely been documented [6]. For *M. leidyi* in particular, the most well studied species among the ctenophores, DVM appears uncommon although few studies have specifically addressed this phenomenon [12]–[14]. Some evidence of DVM has been reported from the Black Sea [15], [16] but the mechanism behind this behavior is unexplored.

*M. leidyi* is a well known invasive species in the Black Sea and European waters, which due to its vast predatory potential has in some cases caused large ecological consequences [17]. While many studies report on *M. leidyi*’s spatial distribution, fewer focus on their vertical spread. The overall vertical distribution pattern of *M. leidyi* was depicted as «somewhat confusing» when reviewed in Mianzan et al. 2010 [18]. While being commonly found above the pycnocline both in its native [12] and exotic [13], [14], [19] habitats, others report high densities near bottom [20] or homogenous vertical distributions [21]. Aggregations also seem to be a common feature, like in the native Pamlico river estuary where individuals were aggregated near surface during day, and dispersed throughout the water column at night [22]. Also in shallow Argentinean waters aggregations were found both in surface and near bottom during daytime [23]. In the Baltic Sea, where *M. leidyi* was introduced recently [24], individuals were on average found above the pycnocline in Skagerrak and Kattegat [25], and below or around the pycnocline in the Central Baltic Proper [25]–[27]. It has been shown that turbulence affects vertical distribution [18], and also low oxygen levels (<1 mg l^−1^) appear to constrain *M. leidyi* vertical distribution [20]. Also predator presence seems to alter *M. leidyi*’s swimming behavior [28].

A range of methods have been used to sample gelatinous plankton, from conventional net sampling [29] to hydro acoustics [10], individual acoustic [9] and non-acoustic tagging [30], filming [8] and visual assessment by divers [16]. While net sampling is the most common method, it only allows a rough vertical resolution. The *in situ* observations obtained from video methods however are well suited for investigating fine scale distribution patterns [8], [31]. Here we used a video-net profiling method to study the fine scale vertical distribution of *M. leidyi* in the Kattegat, the Sound and the central Baltic Sea, a region newly invaded by *M. leidyi*. We describe the vertical distribution in relation to biophysical factors and by filming repeatedly during two 24-hour periods we tested the existence of DVM behavior.

## Methods

### Ethical Statement

No specific permits were required for the described field sampling. The stations visited are not privately owned or protected, and the sampling did not involve endangered or protected species.

### The Study Area

**Figure 1:**
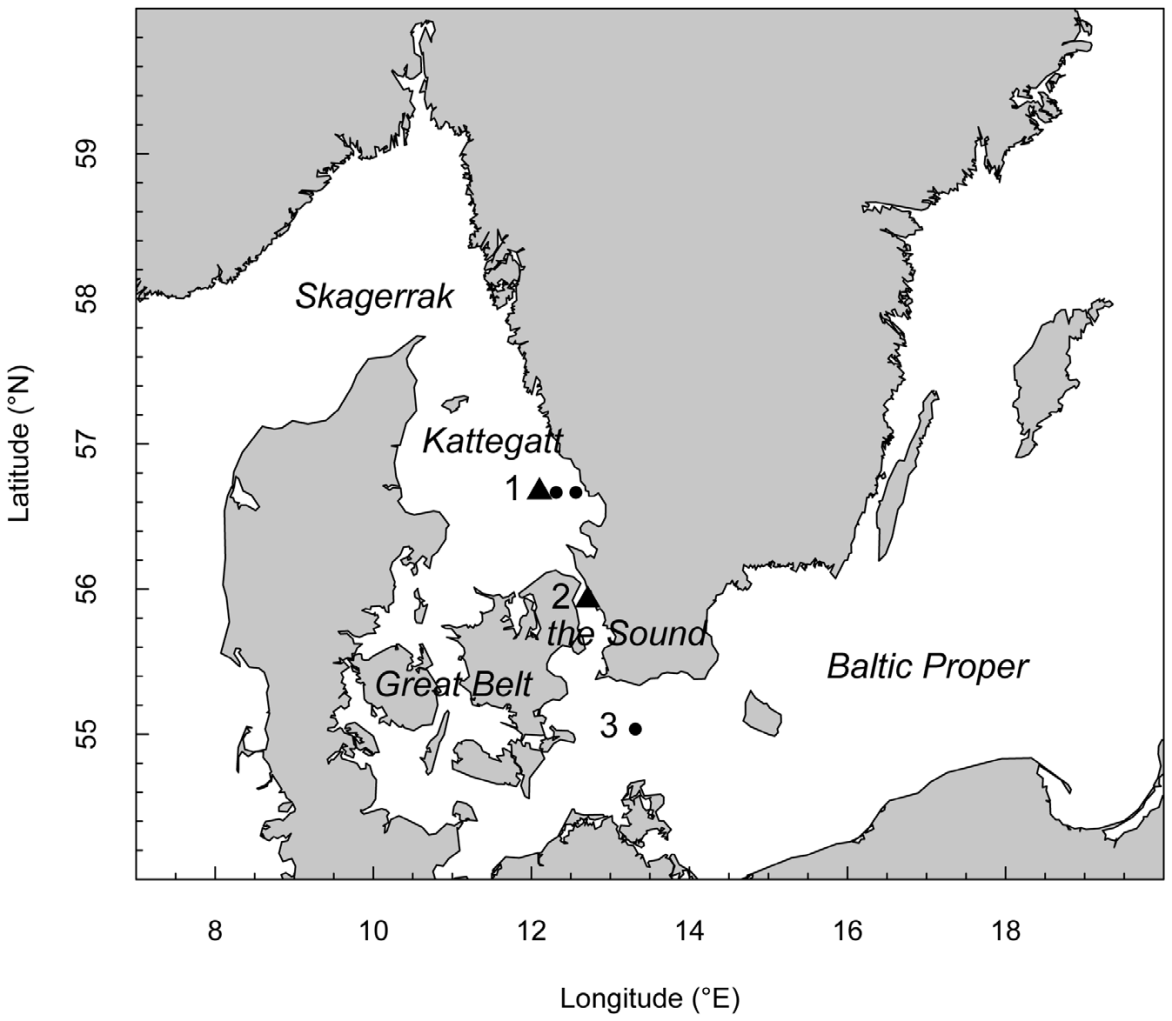
Investigated area. Stations sampled from 13–23 of October 2009 onboard R/V *Skagerak*. Station 1 and 2 (solid triangles) are the 24-hour stations sampled 5 times during a diel cycle, and the dots next to station 1 are the on-shore stations T1 and T2. Station 1 = Anholt, 2 = Ven, and 3 = BY1.

Kattegat is connected to the North Sea and the Baltic Proper through the Great Belt and the Sound (Fig. 1). The two latter are shallow (average 14.6 and 11.7 m respectively) compared to the Kattegat (average 23 m) and the central Baltic Proper (average 62 m) [32]. A pronounced salinity gradient and a permanent stratification characterize the region. Brackish surface water from the Baltic Proper flows northwards and becomes gradually mixed up with the salty and deeper southward flowing water originating from the North Sea. Also the pycnocline is shallower in Kattegat (ca. 15 m) compared to the Baltic Proper (ca. 40–60 m). A temporary thermocline is formed in both seas during spring and summer, which is mixed up and disappears during fall and winter [32]. The limited water exchange into the Baltic Proper [33] contributes to the permanent anoxic layers at greater depths (>50–60 m).

### Sampling Sites

We conducted video-net recordings at 5 locations in Kattegat, the Sound and the Baltic Proper (Fig. 1, Table 1). The sampling was done onboard R/V *Skagerak* from 13^th^ to 23^rd^ of October 2009, which coincided with the peak abundance of *M. leidyi* in 2009 [25]. Two of the stations, Anholt in Kattegat and Ven in the Sound, were sampled every 6^th^ hour during a 24-hour cycle (Anholt A–E, Ven A–E). Anholt was filmed from midday to midday, while Ven from midnight to midnight (Table 1). In close vicinity to the Anholt 24-hour station two additional locations were filmed in direction towards the closest shore (Anholt T1 and T2). The station located in the Baltic Proper, BY1, was only filmed during one occasion. In total 13 video profiles were performed.

**Table 1:** Station and sampling information.

Station information				Video-net			Multinet	
*Name*	*Position (°)*	*Bottom* *depth* *(m)*	*Sampling date* *(DD.MM.YYYY)*	*Deepest* *filmed* *depth (m)*	*Time filmed*	*Count* *(# ind.)*	*Depth intervals (m)*	
							*300 µm* *Ctenophores*	*90 µm* *Zooplankton*
1. Anholt A	56.40 N/12.07 E	55	13.10.2009	36	15∶17–15∶22	537	0-10-20-29	0-10-20-23
Anholt B		55	13.10.2009	41	19∶11–19∶15	572	0-11-20-29	0-10-16-22
Anholt C		52	14.10.2009	40	01∶29–01∶34	819	0-10-20-24	0-9-20-22
Anholt D		55	14.10.2009	40	06∶47–06∶51	636	0-10-20-29	0-4-10-17
Anholt E		55	14.10.2009	41	13∶11–13∶14	352	0-10-20-29	0-9-14-19
Anholt T1	56.40 N/12.19 E	35	14.10.2009	29	15∶42–15∶45	640	0-10-15	0-4-6
Anholt T2	56.40 N/12.34 E	20	14.10.2009	15	18∶03–18∶06	191	0-11	0-14
2. Ven A	55.55 N/12.42 E	43	22.10.2009	35	03∶14–03∶19	25	0-10-20	0-6-9
Ven B		42	22.10.2009	34	07∶51–07∶58	56	0-10-20	0-9-12
Ven C		42	22.10.2009	35	13∶38–13∶43	20	0-5-10-15-20-25	0-5-9-14-19-25
Ven D		42	22.10.2009	35	20∶02–20∶09	46	0-5-10-15-20-25	0-4-9-14-19-24
Ven E		42	23.10.2009	34	01∶25–01∶31	37	0-5-10-15-20-25	0-4-9-14-19-24
3. BY 1	55.02 N/13.18 E	45	21.10.2009	43	13∶57–14∶03	104	0-10-20-28	0-10-20-24

Count is the number of *Mnemiopsis leidyi* observed during downcast. The Multinet columns indicates the depth intervals sampled for the horizontal (300 *µ*m) and vertical (90 *µ*m) tows.

To characterize the water mass during each film-cast, salinity, temperature and oxygen profiles were obtained using a Seabird 911 CTD (Conductivity, Temperature and Depth). Light irradiance was measured with a QSP 2300 spherical PAR (Photosynthetic Active Radiation) sensor from Biospherical Instrument Inc, attached to the CTD.

### Video Recordings

**Figure 2:**
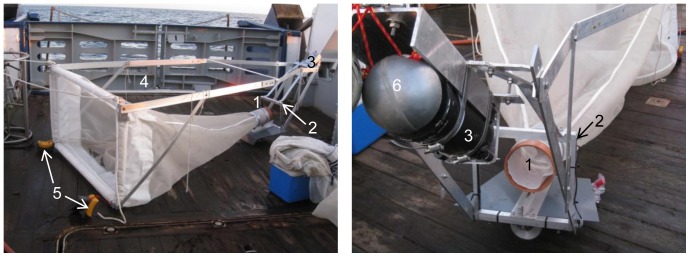
Video-net equipment used for video profiles. 1 = open cylindrical cod end, 2 = light frame, 3 = underwater camera house with a Panasonic SD 100 high definition camera, 4 = position of Scanmar (instrument not shown), 5 = weights, and 6 = floaters. For further details, see text.

We used a combined net and video-frame designed for vertical observations of smaller gelatinous zooplankton such as ctenophores (Fig. 2). The net, with a mouth opening of 1 m^2^, was obliquely towed and directed the net catch through an open cylindrical cod end with a 15 cm inner diameter which was surrounded by a squared light frame with 28 cm long sides covered with a row of four LED (Light Emitting Diodes, 3 W, 55000 K) on the two vertical sides. An underwater battery flask with 12-V lead batteries, mounted on a stabilizing fin below the cod end provided power for the LED illumination. The video-frame combined with a net facilitates the abundance calculations as no visibility distance needs to be calculated [34]. A Panasonic SD 100 high definition video camera in an underwater house was mounted behind and above the frame at a 45° angle and at a distance of 50 cm (Fig. 2). The camera was recording at 50 frames s^−1^, exposure was set to auto and the focus to fixed distance to get sharp records of objects passing through the illuminated frame. The video-net was further equipped with a Scanmar depth sensor to get the real time depth information used to monitor the depth trajectory of the net, and a DST CTD probe to record the depth profiles as well as salinity and temperature, which were later used in the video analyses. The CTD probe was logging every 10 seconds. To improve the orientation of the video-net in the water, two 3 kg weights were attached at each lower corner of the net opening, and two floats were mounted to the camera house to raise the position of the cod end (Fig. 2). During deployment the ship and winch speed were kept constant at approximately 1–1.5 knots and 0.12 m s^−1^ respectively.

### Net Sampling

In connection with the video profiles stratified Multinet samples (0.25 m^−2^ Midi MultiNet, Hydro-bios, Kiel) were taken for morphological species identification of gelatinous zooplankton (300 *µ*m mesh, horizontal tow) and meso zooplankton densities (90 *µ*m mesh, vertical tow) (Table 1). All gelatinous zooplankton from the 300 *µ*m net were directly identified and measured alive on either a transparent backlit table or with the aid of a stereomicroscope. The 90 *µ*m samples were first analyzed for ctenophore larvae, which were individually picked out and dried on cellulose filters at 60°C for 48 h for later genetic species identification. The rest of the sample was then preserved in 4% formaldehyde for later zooplankton identification. Zooplankton densities were converted to biomass using conversion factors from Nielsen and Andersen 2002 [35]. A more detailed description of the Multinet sampling can be found in Haraldsson et al. 2013 [25] for the 300 *µ*m net and in Jaspers et al. 2013 [36] for the 90 *µ*m net and genetic analyzes.

### Video and Data Analyses

Video recordings were converted to MP4 files using Pinnacle Studio 14 (Pinnacle Systems Inc.) and analyzed in VLC media player (version 2, VideoLAN). The films were analyzed frame-by-frame which enabled tracking of individual ctenophores as they passed through the cylindrical cod end. This also reduced the risk of counting an individual more than once. The ctenophores passing through the cod end were all intact showing no visible damage caused by the net. The time of observation and relative size group (small, medium, large) for each *M. leidyi* observation was recorded. Only ctenophores filmed during the downward cast were counted and used in further analyses. The time code from the video analyzes was matched with the time from the DST CTD logging profile to get the depth of occurrence for each ctenophore observation. Small sized *M. leidyi* were defined as ctenophore with no or weakly developed lobes (approx. <14 mm total length based on a subsample of measurements from the video), medium as developed lobated ctenophores, and large as ctenophores with large and well developed lobes (approx. >22 mm total length). The size classes did not correspond directly to a certain developmental stage, but were based on what was easily distinguishable from the videos. While the two larger size classes only contained the lobate stage, the small size class consisted of all developmental stages from tentaculate, transitional to lobate stage. The video and multinet-data are publicly available at the Swedish Meteorological and Hydrological Institute’s database: Svensk Havsarkiv (SHARK).

Other gelatinous zooplankton than the targeted *M. leidyi* were also filmed or caught. *Aurelia aurita* was both filmed and caught in the Sound and Central Baltic Proper, and a few *Cyanea capillata* were caught at Anholt, Bornholm and Gotland deep. *Pleurobrachia pileus* was only filmed and caught in Kattegat. Of the caught gelatinous plankton, *P. pileus* was the only species that could have been classified as *M. leidyi* in the video analyses. If this occurred, the error is minute as the fraction of *P. pileus* of the ctenophores in the Multinet sample never exceeded 1.5%.

Densities (*D*, ind. m^−3^) of *M. leidyi* for the *i*th depth interval (2 m), were calculated by using the time and depth log obtained from DST CTD probe, and the horizontal speed of ship and net-deployment, combined in a simple geometric relationship:
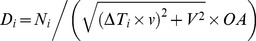
(1)where Δ*T_i_* (s) is the time it takes for the net to travel through the *i*th vertical depth interval, *v* (m s^−1^) is the ship speed minus the speed of the net-deployment during descent, *V* (m) is the vertical depth interval corresponding to 2 m in this study, *OA* (m^2^) is the opening area of the net, and *N_i_* (ind.) is the number of animals counted in the *i*th depth interval. The calculation is similar to that of Båmstedt et al. 2003 [8] except that they used geographic positions instead of time and speed.

Further, the mean depth (*Z_m_*, m) and standard deviation (*Z_s_*, m) of the *M. leidyi* vertical distribution were calculated according to Dupont and Aksnes 2012 [37]:
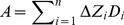
(2)

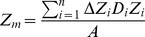
(3)

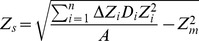
(4)where A is the integrated abundance (ind. m^−2^), *D_i_* represents the mean *M. leidyi* concentration (ind. m^−3^) of the sampled depth layer Δ*Z_i_*, *Z_i_* is the mid-depth of each layer *i* which were set to every 2 m filmed, and *n* is the number of depth layers.

Finally, the overlap coefficients (*OC*) for each separate size group of *M. leidyi* (*M*) in relation to zooplankton (*Z*) were calculated according to Horn 1966 [38]:
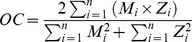
(5)where *n* is the number of depth strata covering the water column, *M* and *Z* are the relative abundances of predator and prey, respectively, per *i*th depth strata. A value of 1 indicates full overlap, while 0 indicates no overlap. As the vertical sampling resolution differed between the video-net and Multinet method, the zooplankton depth intervals given by the Multinet (Table 1) were used also in the *M. leidyi* calculation.

The hypothesis that *M. leidyi* did not migrate was tested using linear regressions. Mean depth (*Z_m_*) for each size class was defined as the dependent variable and time from the solar noon as the independent variable. The time of solar noon for each filming location and occasion was taken from NOAA’s (National Oceanic and Atmospheric Administration) solar calculator (http://www.esrl.noaa.gov/gmd/grad/solcalc/, assessed December 2012), and gives a proxy of the approximate light intensity. The calculator accounts for the geographical location (i.e. latitude and longitude) and the date when calculating the time of solar noon. “Central European time zone” and “daytime saving time” (DST) were used in the settings of the calculator.

## Results

### Hydrography

**Figure 3:**
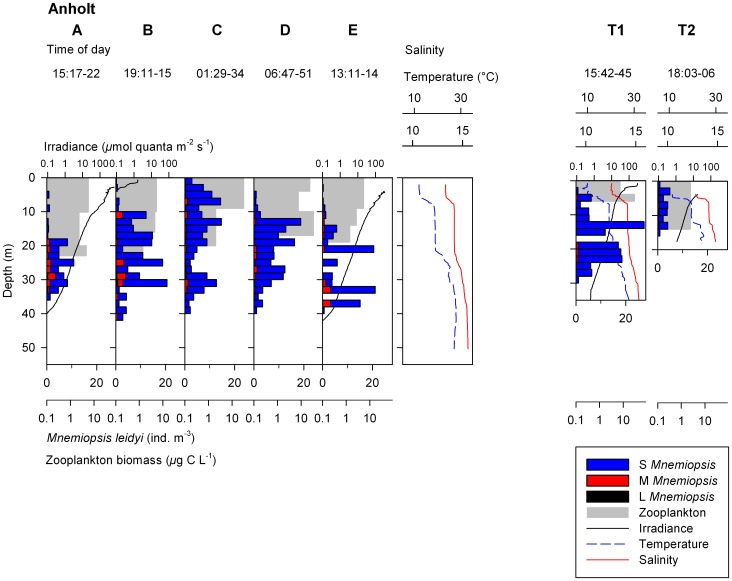
Depth profiles of *Mnemiopsis leidyi*. Vertical distribution and densities (ind. m^−3^) for three size classes (S = Small, M = Medium, L = Large) of *M. leidyi* and zooplankton at Anholt station in Kattegat during 24-h and the on-shore stations Anholt T1 and T2 together with biophysical variables. Station codes are given in Table 1. Irradiance and zooplankton biomass are plotted on log scale.

**Figure 4:**
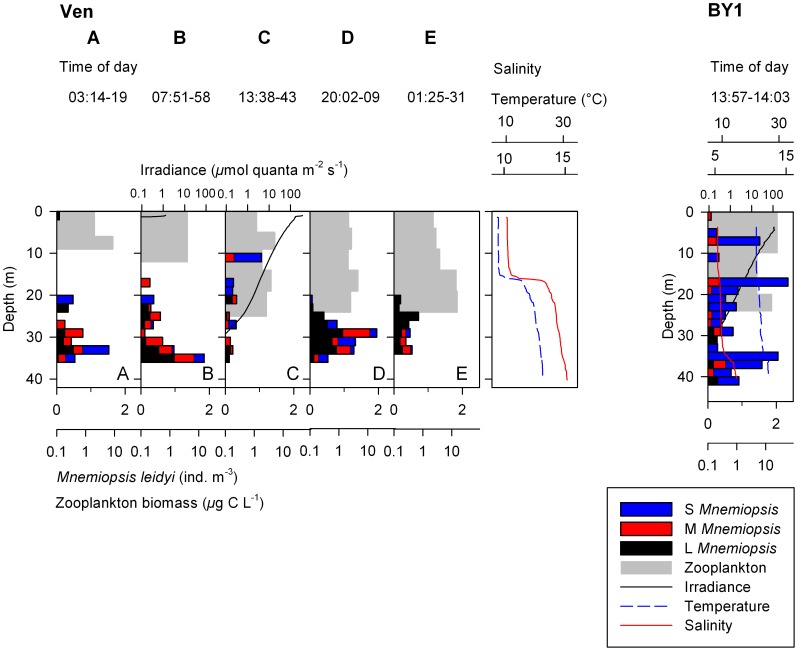
Depth profiles of *Mnemiopsis leidyi*. Vertical distribution and densities (ind. m^−3^) for three size classes (S = Small, M = Medium, L = Large) of *M. leidyi* and zooplankton at Ven station in the Sound during 24-h, and the single station in the Central Baltic Sea (BY1) together with biophysical variables. Irradiance and zooplankton biomass are plotted on log scale.

The thermocline and halocline coincided throughout the investigated area (Fig. 3 and 4). Two clear pycnoclines (at 6 and 22 m depth respectively) were present at Anholt (Fig. 3), and one at Ven at 14 m depth (Fig. 4). Also BY1 had two pycnoclines, although less pronounced (Fig. 4). The water was well oxygenated from surface to bottom and concentrations never went below 1.2 ml l^−1^.

### Vertical Distribution and Migration

#### 24-hour stations

**Figure 5:**
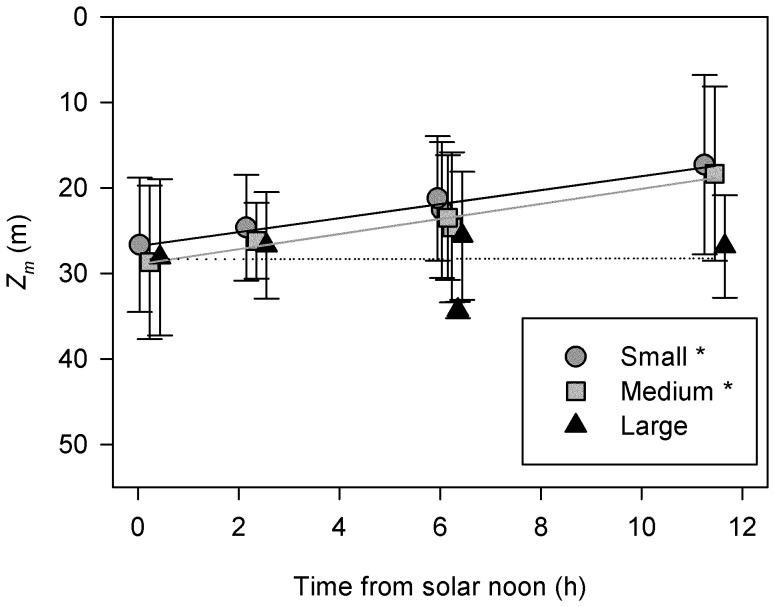
Test of the hypothesis of no migration. Weighted mean depth *Z_m_* of small, medium and large *Mnemiopsis leidyi* as a function of time from solar noon at Anholt 24-hour station, where the latter was used as a proxy for the daily variation in surface light intensity. Error bars are the spread *Z_s_* (m) around the mean depth. The lines are the linear regressions (Table 2), where the asterisk indicates statistical significance. The symbols are slightly shifted to facilitate readability.

The hypothesis of no migration was rejected for the small and medium sized individuals at the Anholt station as the slopes of the regressions were statistically different from zero and indicated migration speeds of 0.82 and 0.88 m h^−1^, respectively (Fig. 5, Table 2). This corresponds to a total vertical migration of approximately 10 m from solar noon to midnight. The observations for the large individuals, however, were consistent with no migration and they were centered at a depth of 28 m (Fig. 5). Similarly, no migration was detected for the zooplankton, which centered around 10 m depth (Table 2). Small individuals dominated at the Anholt station (89%), followed by medium (9.5%) and large sized (1.5%) *M. leidyi* (table 3).

**Table 2:** Test of the hypothesis of no migration.

Group	*a* (m)	*b* (m h^−1^)	R^2^	*p*	n
Small *M. leidyi*	26.82 (0.47)	−0.82 (0.07)	0.98	0.002	5
Medium *M. leidyi*	28.93 (0.64)	−0.88 (0.10)	0.96	0.003	5
Large *M. leidyi*	28.38 (3.03)	−0.01 (0.46)	0.00	0.982	5
ZooplanktonAnholt	10.28 (0.96)	−0.35 (0.15)	0.65	0.098	5
Zooplankton Ven	9.67 (4.98)	0.20 (0.64)	0.03	0.790	5

Regression equations (*y = bx+a*) describing the relationship between y, which is mean depth (*Z_m_*, m) of three size classes of *Mnemiopsis leidyi* at the Anholt station and zooplankton at both Anholt and Ven station, and x, the time (h) from solar noon. Values within parenthesis is the standard error of the coefficient, R^2^ is the coefficient of determination, *p* the significance level, and n the number of values in the analyses. The expectation of no migration corresponds to a b-value not different from zero. Negative values of *b* indicate movement towards the surface between solar noon and midnight.

**Table 3:** Density estimates of *Mnemiopsis leidyi* from video-net and Multinet.

Station	*Volume filmed* Video-net (m^3^)	*Abundance* Video-net (ind. m^−3^)				*Abundance*Multinet(ind. m^−3^)	*Z_m_* (*Z_s_*)Video-net(m)	*Z_m_* (*Z_s_*)Multinet(m)
		All	Small	Medium	Large	All		
Anholt A	136.8	3.26	2.54	0.58	0.13	5.92	25.0 (6.0)	10.6 (6.5)
Anholt B	110.2	6.74	5.87	0.70	0.17	1.91	22.9 (8.1)	7.3 (5.2)
Anholt C	133.7	6.93	6.47	0.41	0.05	5.01	17.4 (10.5)	8.6 (6.1)
Anholt D	119.5	6.40	5.98	0.40	0.01	11.14	21.4 (7.3)	8.3 (4.9)
Anholt E	134.4	4.25	3.68	0.53	0.04	11.03	26.9 (8.0)	10.7 (5.3)
Anholt mean (SD)		5.52 (1.66)				7.00 (4.01)		
Ven A	121.2	0.23	0.08	0.08	0.08	0.42	28.9 (5.9)	11.8 (4.7)
Ven B	158.7	0.23	0.03	0.11	0.09	2.45	28.1 (5.2)	14.9 (0.9)
Ven C	126.9	0.16	0.09	0.04	0.03	0.68	19.4 (7.9)	18.5 (4.0)
Ven D	155.4	0.35	0.07	0.09	0.20	1.38	29.7 (2.7)	20.9 (3.5)
Ven E	160.9	0.17	0.01	0.03	0.13	1.56	27.7 (3.6)	18.9 (6.1)
Ven mean (SD)		0.23 (0.08)				1.30 (0.80)		
Anholt T1	132.3	8.53	8.24	0.22	0.07	13.42	17.2 (5.9)	9.6 (4.1)
Anholt T2	74.9	3.03	2.67	0.32	0.04	5.53	7.6 (3.8)	5.3 (na)
BY1	149.7	0.68	0.55	0.08	0.05	0.69	25.6 (11.2)	14.7 (8.0)

*Z_m_* is mean depth and *Z_s_* is spread (Eqs. 3 and 4). SD is standard deviation.

Observations from the video-net showed that the bulk of all ctenophores (71 and 59%) resided below the deepest pycnocline at 22 m around midday, which was filmed twice during the 24-h cycle (Fig. 3). The opposite situation was found during midnight when most ctenophores (67%) were found above 22 m. Only during midnight were animals observed in the surface layer above the shallow pycnocline at around 6 m depth, co-occurring with low irradiance and peak zooplankton biomass (Fig. 3). At midnight, 15% of the individuals resided in the surface layer while only 1% was found here during daytime.

At Ven, almost all ctenophores (98–100% at respective filming occasion) were found in the saline water below the pycnocline, except at noon when 4 individuals (corresponding to 39% of all observed individuals at this occasion) were found above the pycnocline (Fig. 4). Except for these 4 individuals, no vertical migration could be detected, and no regression model was applied due to the low numbers. Further, of all observed individuals at Ven, large (46%) or medium (30%) sizes dominated (table 3).

#### Individual stations

After sampling the Anholt 24-hour station additional stations closer to shore were sampled. The densities were highest at Anholt T1 (8.53 ind. m^−3^), and lower at station Anholt T2 (3.03 ind. m^−3^) which was the location closest to shore (Fig. 3). The high density station (Anholt T1) had highest ctenophore densities below the pycnocline. The low density station (Anholt T2), which was sampled during early evening, had similar densities above and below the pycnocline (Fig. 3).

In the Baltic Proper at station BY1, *M. leidyi* was found in significant numbers with densities of 0.68 ind. m^−3^ (Table 3, Fig. 4). Ctenophores were found at all depths, and of all size classes only the large individuals had a mean depth (Z_m_, 35 m) below the pycnocline at 34 m depth.

### *M. leidyi* in Relation to Environmental Factors

**Figure 6:**
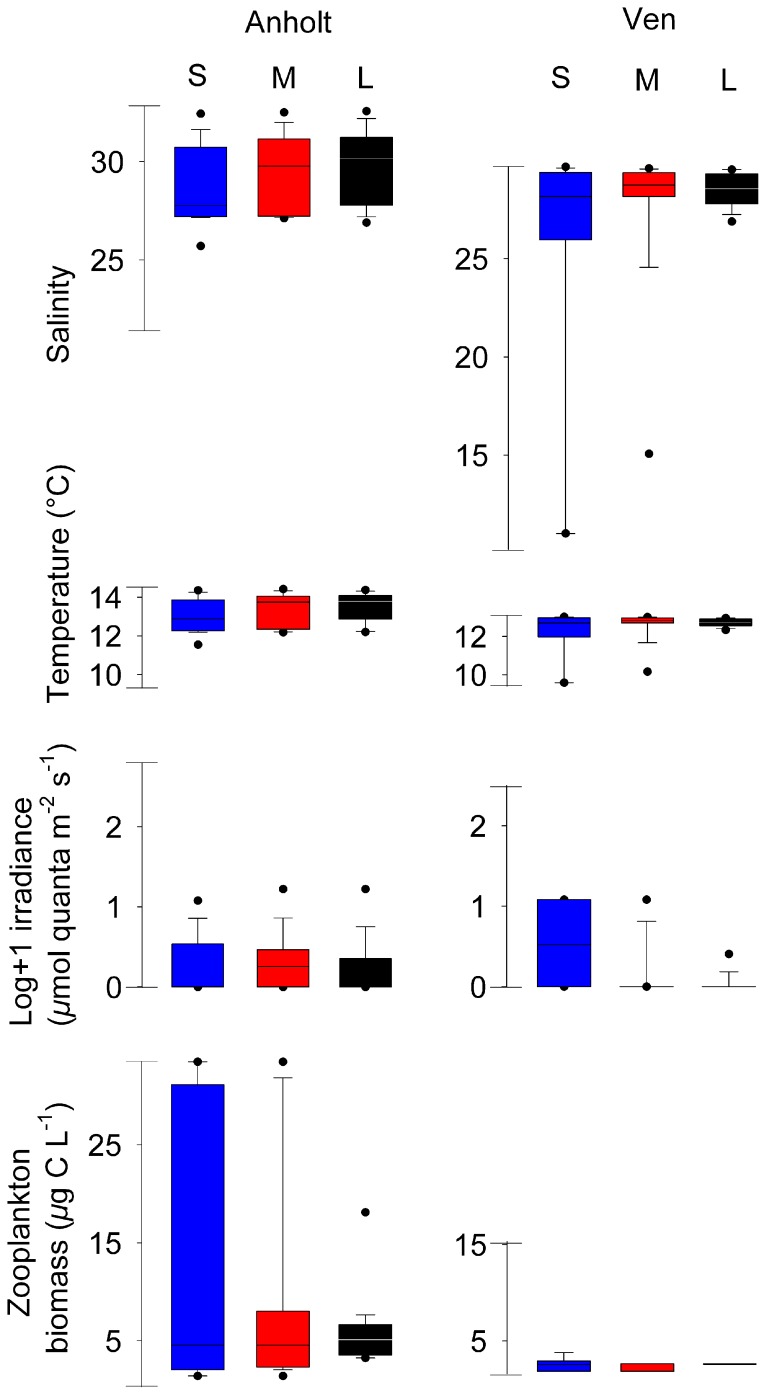
Observed environmental range. Distributions of environmental variables at Anholt and at Ven for three separate size classes of *Mnemiopsis leidyi*. Irradiance was only measured during daytime. The box represents 50% of all observations, with the solid line representing the median, the whiskers 10^th^ and 90^th^ percentile, and the dots the 5^th^ and 95^th^ percentile. Axes correspond to minimum and maximum of measured variables at the respective location. S = Small, M = Medium, L = Large. Small and medium individuals in Kattegat migrated.

Most observations of all size classes at the two 24-hour stations were associated with high salinity (Fig. 6). 90% (up to 95% at Anholt ) of *M. leidyi* were found at salinities >25 with exception for small individuals at Ven where only 75% were found above 25. At Anholt station only the migrating individuals found in the top 10 m were found in salinities below this threshold. Also common for all individuals were an apparent preference for low irradiance levels, with 90% of all individuals found at irradiance levels <11 µmol quanta m^−2^ s^−1^, which was well below the maximum irradiance level measured (661 and 365 µmol quanta m^−2^ s^−1^ at Anholt and Ven respectively). Irradiance and salinity were negatively correlated (Spearman’s rank correlation Anholt; *ρ* = −0.56, *p*<<0.01, n = 461; Ven: *ρ* = −0.93, *p*<<0.01, n = 318). Also temperature was strongly correlated with salinity (Anholt and Ven; *ρ* = 0.97, *p*<<0.01, n = 4065). *M. leidyi* showed an apparent preference for higher temperatures, although the range of temperatures was only over a few degrees (Fig. 6).

**Figure 7:**
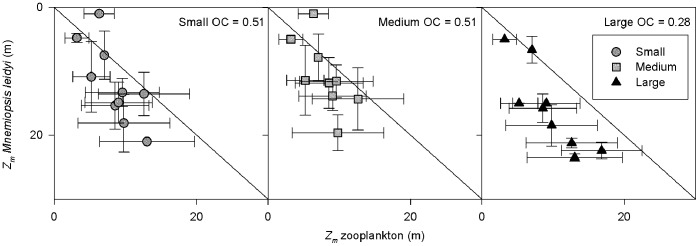
Predator and prey overlap. Weighted mean depth *Z_m_* (error bars *Z_s_*) of three size classes of *Mnemiopsis leidyi* plotted against the weighted mean depth of their potential zooplankton prey. OC = overlap coefficient (averaged over all stations) where a value of 1 indicates full vertical overlap, and 0 no vertical overlap. *M. leidyi* mean depth is based on the same maximum depth as the zooplankton.

Small and medium sized migrating individuals overlapped with zooplankton to a larger extent than the non-migrating largest size class as indicated by the larger OC (Fig. 7), although *M. leidyi* typically resided deeper than the bulk of zooplankton. Further, the migrating individuals were on average found at higher oxygen levels, which was likely a consequence of increasing oxygen levels towards the surface.

## Discussion

Our observations suggest that *M. leidyi* is able to perform DVM. The migration distance based on the regression analyses was on average >10 m for the two smallest size classes, while the largest size class seemed to stay deeper without migrating. However, DVM was not detected at all locations, which suggest that DVM in *M. leidyi* is controlled by several factors. Light is a common cue for migration among zooplankton [4], and this might also apply to *M. leidyi*. In our observations, both migrating and non-migrating individuals appeared to avoid irradiance levels >11 µmol quanta m^−2^ s^−1^ (Fig. 6). Also the significant regression between mean depth and time of day suggests that their vertical position might depend on light level (Fig. 5), which has been shown for scyphozoan jellyfishes [39]. Only the migrating individuals encountered the highest zooplankton biomass (Fig. 6), implying larger food availability for the migrating compared to the non migrating individuals.

The light sensitivity of ctenophores is debated as light sensing organs have not been identified within the phylum [6]. However, spawning in *M. leidyi* takes place a few hours after sunset, and modification of the light environment is therefore a standard lab procedure to activate spawning in cultured animals [40]. In addition, the photocytes that are involved in light production in ctenophores may also possess light sensing functions [41]. Thus sensitivity to light cannot be ruled out as one of the factors governing DVM in *M. leidyi*. If *M. leidyi* are able to regulate their vertical distribution in response to light, this implies that water clarity can have a strong effect on the vertical distribution, similarly to what has been found for the deep sea scyphozoan *Periphylla periphylla* [42].

While light is generally considered to be a proximate factor for DVM, the ultimate factor for the evolution of DVM is considered to be predator avoidance [2]. During daytime when the surface layers are illuminated, the visibility increases and also the risk of being detected by visual predators. Several reviews and studies highlight that fish as predators on jellyfish is a neglected area [43]–[46]. Various fish are indeed known to feed on *M. leidyi* [47], [48]. In Scandinavian waters several potential visual predators on *M. leidyi* exist. Planktivorous fish may generally feed on ctenophores [45], for example, mackerel (*Scomber scombrus*) prey on gelatinous plankton independent of the presence of alternative zooplankton prey [49]. Also the lumpfish *Cyclopterus lumpus* is a known predator of gelatinous plankton [50]. Other non visually feeding gelatinous plankton are also known to be important predators of *M. leidyi* [51]–[54], and may in some cases control *M. leidyi* populations [55].

Although DVM can be very persistent within some populations or regions [56], the behavior is often described as flexible, varying with e.g. predator presence, season and ontogenetic stage [57], [58]. Ontogenetic differences in migratory behavior, similar to what we have found, have been described for other species. The deep sea scyphomedusae *Periphylla periphylla*, for example, shows different activity and migration behavior depending on size [10], [34]. Also among the planktivorous fish, *Maurolicus muelleri*, DVM differs between ontogenetic stages and this has been related to the predation risk [59], [60]. Theoretical models suggest different optimal strategies between growth and survival depending on life stage, where small individuals tolerate higher predation risk in shallower and more illuminated water in order to achieve sufficient feeding and growth rates [59], [61]. The apparent size dependent migration in our study is possibly due to similar life stage dependent strategies. While occasional reproduction has been described in larval *Mnemiopsis* [62], continuous reproduction starts at the lobate stage approximately >6.5 mm [63] and egg production increases with body size [64], [65]. The large individuals with the highest reproductive potential were residing deep without signs of migration towards surface. According to previous studies [65] [66] their reproductive success were likely favored by the higher salinities and slightly warmer temperatures found at depth.

Too low salinity appears to be a major factor limiting the population expansion in the Baltic Proper [25], [65]. Avoidance of low salinities was also indicated by the vertical distributions in the present study (Fig. 6). We suggest that low salinity may prohibit migration in strongly stratified waters such as seen in the Sound. A sharp halocline was common to all locations where the bulk of the individuals resided below the halocline (i.e. Anholt T2 and Ven). Gelatinous plankton maintains the same osmolarity as the surrounding seawater [67], [68], and their ability of osmotic accommodation constrains movement through salinity discontinuities [69]. Strong salinity stratification may therefore act as a physical barrier.

The fine scale vertical resolution obtained with the video-net method enabled the detection of DVM behavior in *M. leidyi*, which would have stayed unrecognized with the Multinet sampling (Table 3). Previous studies reporting on *M. leidyi*’s vertical distribution are commonly based on net sampling with a vertical sampling resolution of 10 meters or more [70], [71]. This depth resolution is larger than the maximum migration amplitude observed in this study (i.e. 10 m), and indicates that previous studies could have missed a potential DVM behavior due to the sampling technique. Indeed, Hays et al. (2012) [72] used novel techniques (tagging) and were able to show vertical migration in medusa jellyfish which would not have been detected with traditional net sampling. However, the low number of stations sampled in this study limits the understanding of how extensive and frequent this behavior may be. We encourage researchers to consider the possibility of DVM behavior in future studies of *M. leidyi*. Further, the estimated mean depth was deeper for the video-net profile than for the Multinet (Table 3), but it had also a deeper maximum sampling depth (Table 1). Densities obtained from the video-net profile were lower than for the Multinet (Table 3, pairwise t-test on log transformed data: t = −3.758, df = 17, *p* = 0.02). The video-net density estimate has an uncertainty due to approximate ship and winch speeds used in the calculations. However, we consider the relative abundance at a given location reliable since both ship and winch speeds were kept constant during the video-net tow. This error might have contributed to the difference in density estimates obtained by video-net and the Multinet (Table 3). In a similar way, the different sampling resolution and maximum sampling depth between the two methods may also have limited the comparison with potential zooplankton prey.

In conclusion, our data suggests the existence of DVM in *M. leidyi*. This migration pattern was associated with the younger life stages, but appeared to be constrained in locations with strong haloclines. The DVM pattern was characterized by smaller individuals that approached the zooplankton-rich surface layer only at nighttime, while the large individuals seemed to stay below this layer at all times. Both for the migrating and non-migrating *M. leidyi*, the vertical distributions are consistent with avoidance of high irradiance levels, which may reflect avoidance from visual predators. Proximate control of the vertical distribution as our study suggests, could potentially be modeled with a proximate model. Such a model could be that *M. leidyi* moves according to random walk in the depth layer with prey densities above a given threshold, but where the random walk is constrained by: *i)* avoidance of light intensities larger than a given threshold, *ii)* avoidance of low salinity and salinity gradients larger than a given threshold, and *iii)* where adult *M. leidyi* prefer high temperatures over high prey density to increase fecundity.

## References

[1] McManus MA, Woodson CB (2012) Plankton distribution and ocean dispersal. J Exp Biol 215: 1008–1016. 22357594 10.1242/jeb.059014

[2] Hays GC (2003) A review of the adaptive significance and ecosystem consequences of zooplankton diel vertical migrations. Hydrobiologia 503: 163–170.

[3] Ringelberg J (1995) Changes in light-intensity and diel vertical migration – A comparison of marine and fresh-water environments. J Mar Biol Assoc U.K. 75: 15–25.

[4] Cohen JH, Forward RB Jr (2009) Zooplanktion diel vertical migration – A review of proximate control. In: Oceanography and Marine Biology: An Annual Review, Vol Gibson RN, Atkinson RJA, Gordon JDM, editors. 47: 77–109.

[5] Haney JF (1988) Diel patterns of zooplankton behavior. Bull Mar Sci 43: 583–603.

[6] Graham WM, Pages F, Hamner WM (2001) A physical context for gelatinous zooplankton aggregations: a review. Hydrobiologia 451: 199–212.

[7] Schuyler Q, Sullivan BK (1997) Light responses and diel migration of the scyphomedusa *Chrysaora quinquecirrha* in mesocosms. J Plankton Res 19: 1417–1428.

[8] Båmstedt U, Kaartvedt S, Youngbluth M (2003) An evaluation of acoustic and video methods to estimate the abundance and vertical distribution of jellyfish. J Plankton Res 25: 1307–1318.

[9] Moriarty PE, Andrews KS, Harvey CJ, Kawase M (2012) Vertical and horizontal movement patterns of scyphozoan jellyfish in a fjord-like estuary. Mar Ecol Prog Ser 455: 1–12.

[10] Kaartvedt S, Titelman J, Rostad A, Klevjer TA (2011) Beyond the average: Diverse individual migration patterns in a population of mesopelagic jellyfish. Limnol Oceanogr 56: 2189–2199.

[11] Dupont N, Klevjer TA, Kaartvedt S, Aksnes DL (2009) Diel vertical migration of the deep-water jellyfish *Periphylla periphylla* simulated as individual responses to absolute light intensity. Limnol Oceanogr 54: 1765–1775.

[12] Purcell JE, Nemazie DA, Dorsey SE, Houde ED, Gamble JC (1994) Predation mortality of bay Anchovy *Anchoa mitchilli* eggs and larvae due to scyphomedusae and ctenophores in Chesapeak Bay. Mar Ecol Prog Ser 114: 47–58.

[13] Mutlu E (1999) Distribution and abundance of ctenophores and their zooplankton food in the Black Sea. II. *Mnemiopsis leidyi* . Mar Biol 135: 603–613.

[14] Kideys AE, Romanova Z (2001) Distribution of gelatinous macrozooplankton in the southern Black Sea during 1996–1999. Mar Biol 139: 535–547.

[15] Zaika VE, Sergeeva NG (1991) Diurnal changes of the population structure and vertical distribution of *Mnemiopsis mccradyi* in the Black Sea. Hydrobiol J 27: 15–19.

[16] Vereshchaka AL (2002) Features of the microscale distribution of the gelatinous macroplankton in the Black Sea off Gelendzhik (August 2000). Oceanology 42: 83–90.

[17] Kideys AE (2002) Fall and rise of the Black Sea ecosystem. Science 297: 1482–1484. 12202806 10.1126/science.1073002

[18] Mianzan HW, Martos P, Costello JH, Guerrero RA (2010) Avoidance of hydrodynamically mixed environments by *Mnemiopsis leidyi* (Ctenophora: Lobata) in open-sea populations from Patagonia, Argentina. Hydrobiologia 645: 113–124.

[19] Shiganova TA, Mirzoyan ZA, Studenikina EA, Volovik SP, Siokou-Frangou I, et al (2001) Population development of the invader ctenophore *Mnemiopsis leidyi*, in the Black Sea and in other seas of the Mediterranean basin. Mar Biol 139: 431–445.

[20] Keister JE, Houde ED, Breitburg DL (2000) Effects of bottom-layer hypoxia on abundances and depth distributions of organisms in Patuxent River, Chesapeake Bay. Mar Ecol Prog Ser 205: 43–59.

[21] Kremer P, Nixon S (1976) Distribution and abundance of ctenophore, *Mnemiopsis leidyi* in Narragansett Bay. Estuar Coast Mar Sci 4: 627–639.

[22] Miller RJ (1974) Distribution and biomass of an estuarine ctenophore population, *Mnemiopsis leidyi* (A. Agassiz). Chesap Sci 15: 1–8.

[23] Costello JH, Mianzan HW (2003) Sampling field distributions of *Mnemiopsis leidyi* (Ctenophora, Lobata): planktonic or benthic methods? J Plankton Res 25: 455–459.

[24] Javidpour J, Sommer U, Shiganova TA (2006) First record of *Mnemiopsis leidyi* A. Agassiz 1865 in the Baltic Sea. Aquat Invasions 1: 299–302.

[25] Haraldsson M, Jaspers C, Tiselius P, Aksnes DL, Andersen T, et al (2013) Environmental constraints of the invasive *Mnemiopsis leidyi* in Scandinavian waters. Limnol Oceanogr 58: 763–763.

[26] Huwer B, Storr-Paulsen M, Riisgård HU, Haslob H (2008) Abundance, horizontal and vertical distribution of the invasive ctenophore *Mnemiopsis leidyi* in the central Baltic Sea, November 2007. Aquat Invasions 3: 113–124.

[27] Schaber M, Haslob H, Huwer B, Harjes A, Hinrichsen HH, et al (2011) Spatio-temporal overlap of the alien invasive ctenophore *Mnemiopsis leidyi* and ichthyoplankton in the Bornholm Basin (Baltic Sea). Biol Invasions 13: 2647–2660.

[28] Titelman J, Hansson LJ, Nilsen T, Colin SP, Costello JH (2012) Predator-induced vertical behavior of a ctenophore. Hydrobiologia 690: 181–187.

[29] Purcell JE (2009) Extension of methods for jellyfish and ctenophore trophic ecology to large-scale research. Hydrobiologia 616: 23–50.

[30] Hays GC, Doyle TK, Houghton JDR, Lilley MKS, Metcalfe JD, et al (2008) Diving behaviour of jellyfish equipped with electronic tags. J Plankton Res 30: 325–331.

[31] Graham WM, Martin DL, Martin JC (2003) In situ quantification and analysis of large jellyfish using a novel video profiler. Mar Ecol Prog Ser 254: 129–140.

[32] Fonselius S (1996) Västerhavets och Östersjöns oceanografi. Swedish Meteorological and Hydrological Institute, Västra Frölunda. 15 p, 49 p and 52 p.

[33] Sayin E, Krauss W (1996) A numerical study of the water exchange through the Danish Straits. Tellus 48A: 324–341.

[34] Youngbluth MJ, Båmstedt U (2001) Distribution, abundance, behavior and metabolism of *Periphylla periphylla*, a mesopelagic coronate medusa in a Norwegian fjord. Hydrobiologia 451: 321–333.

[35] Nielsen TG, Andersen CM (2002) Plankton community structure and production along a freshwater-influenced Norwegian fjord system. Mar Biol 141: 707–724.

[36] Jaspers C, Haraldsson M, Lombard F, Bolte S, Kiørboe T (2013) Seasonal dynamics of early life stages of invasive and native ctenophores give clues to invasion and bloom potential in the Baltic Sea. J Plankton Res 35: 582–594.

[37] Dupont N, Aksnes DL (2012) Effects of bottom depth and water clarity on the vertical distribution of *Calanus spp* . J Plankton Res 34: 263–266.

[38] Horn HS (1966) Measurement of overlap in comparative ecological studies. Am Nat 100: 419–424.

[39] Ferraris M, Berline L, Lombard F, Guidi L, Elineau A, et al (2012) Distribution of *Pelagia noctiluca* (Cnidaria, Scyphozoa) in the Ligurian Sea (NW Mediterranean Sea). J Plankton Res 34: 874–885.

[40] Pang K, Martindale MQ (2008) *Mnemiopsis leidyi* spawning and embryo collection. Cold Spring Harb Protoc (2008) doi:10.1101/pdb.emo106. 10.1101/pdb.prot5085 21356725

[41] Schnitzler CE, Pang K, Powers ML, Reitzel AM, Ryan JF, et al. (2012) Genomic organization, evolution, and expression of photoprotein and opsin genes in *Mnemiopsis leidyi*: a new view of ctenophore photocytes. BMC Biol 10:107. Available: http://www.biomedcentral.com/1741-7007/10/107 Accessed 2013 Mar 20. 10.1186/1741-7007-10-107 PMC3570280 23259493

[42] Sørnes TA, Aksnes DL, Båmstedt U, Youngbluth MJ (2006) Causes for mass occurrences of the jellyfish *Periphylla periphylla*: a hypothesis that involves optically conditionoed retention. J Plankton Res 29: 157–167.

[43] Ates RML (1988) Medusivorous fishes, a review. Zoologische Mededelingen (Leiden) 62: 29–42.

[44] Purcell JE, Arai MN (2001) Interactions of pelagic cnidarians and ctenophores with fish: a review. Hydrobiologia 451: 27–44.

[45] Arai MN (2005) Predation on pelagic coelenterates: a review. J Mar Biol Assoc U.K. 85: 523–536.

[46] Cardona L, Alvarez de Quevedo I, Borrell A, Aguilar A (2012) Massive consumption of gelatinous plankton by Mediterranean apex predators. PLoS ONE 7(3): e31329. 22470416 10.1371/journal.pone.0031329 PMC3310041

[47] Oviatt CA, Kremer PM (1977) Predation on the ctenophore, *Mnemiopsis leidyi*, by Butterfish, *Peprilus triacanthus*, in Narragansett Bay, Rhode Island. Chesapeake Science 18: 236–240.

[48] Mianzan HW, Mari N, Prenski B, Sanchez F (1996) Fish predation on neritic ctenophores from the Argentine continental shelf: A neglected food resource? Fish Res 27: 69–79.

[49] Runge JA, Pepin P, Silvert W (1987) Feeding-behavior of the Atlantic Mackerel *Scromber scombrus* on the hydromedusa *Aglantha digitale* . Mar Biol 94: 329–333.

[50] Swedish Agency for Marine and Water Management (2010) Fiskebestånd och miljö i hav och sötvatten. Resurs- och miljööversikt 2010. Fiskeriverket. Available: www.havochvatten.se.

[51] Purcell JE, Cowan JH (1995) Predation by the scyphomedusan *Chrysaora quinquecirrha* on *Mnemiopsis leidyi* ctenophores. Mar Ecol Prog Ser 129: 63–70.

[52] Hosia A, Titelman J (2011) Intraguild predation between the native North Sea jellyfish *Cyanea capillata* and the invasive ctenophore *Mnemiopsis leidyi* . J Plankton Res 33: 535–540.21984852

[53] Hosia A, Titelman J, Hansson LJ, Haraldsson M (2011) Interactions between native and alien ctenophores: *Beroe gracilis* and *Mnemiopsis leidyi* in Gullmarsfjorden. Mar Ecol Prog Ser 422: 129–138.

[54] Tilves U, Purcell JE, Marambio M, Canepa A, Olariaga A, Fuentes V (2013) Predation by the scyphozoan Pelagia noctiluca on *Mnemiopsis leidyi* ctenophores in the NW Mediterranean Sea. J Plankton Res 35: 218–224.

[55] Condon RH, Steinberg DK (2008) Development, biological regulation, and fate of ctenophore blooms in the York River estuary, Chesapeake Bay. Mar Ecol Prog Ser 369: 153–168.

[56] Valle-Levinson A, Castro AT, de Velasco GG, Armas RG (2004) Diurnal vertical motions over a seamount of the southern Gulf of California. J Mar Syst 50: 61–77.

[57] Ohman MD (1990) The demographic benefits of diel vertical migration by zooplankton. Ecol Monogr 60: 257–281.

[58] Hays GC (1995) Ontogenetic and seasonal variation in the diel vertical migration of the copepods *Metridia lucens* and *Metridia longa* . Limnol Oceanogr 40: 1461–1465.

[59] Giske J, Aksnes DL (1992) Ontogeny, season and trade-offs: Vertical distribution of mesopelagic fish *Maurolicus muelleri* . Sarsia 77: 253–261.

[60] Staby A, Srisomwong J, Rosland R (2013) Variation in DVM behaviour of juvenile and adult pearlside (*Maurolicus muelleri*) linked to feeding strategies and related predation risk. Fish Oceanogr 22: 90–101.

[61] Rosland R, Giske J (1997) A dynamic model for the life history of *Maurolicus muelleri*, a pelagic planktivorous fish. Fish Oceanogr 6: 19–34.

[62] Martindale MQ (1987) Larval reproduction in the ctenophore *Mnemiopsis maccradyi* (order lobata). Mar Biol 94: 409–414.

[63] Jaspers C (2012) Ecology of gelatinous plankton. With emphasis on feeding interactions, distribution patterns and reproduction biology of *Mnemiopsis leidyi* in the Baltic Sea. PhD dissertation, Copenhagen.

[64] Finenko GA, Kideys AE, Anninsky BE, Shiganova TA, Roohi A, et al (2006) Invasive ctenophore *Mnemiopsis leidyi* in the Caspian Sea: feeding, respiration, reproduction and predatory impact on the zooplankton community. Mar Ecol Prog Ser 314: 171–185.

[65] Jaspers C, Møller LF, Kiørboe T (2011) Salinity gradient of the Baltic Sea limits the reproduction and population expansion of the newly invaded comb jelly *Mnemiopsis leidyi* . PLoS ONE 6(8): e24065. 21887373 10.1371/journal.pone.0024065 PMC3162597

[66] Costello JH, Sullivan BK, Gifford DJ, Van Keuren D, Sullivan LJ (2006) Seasonal refugia, shoreward thermal amplification, and metapopulation dynamics of the ctenophore *Mnemiopsis leidyi* in Narragansett Bay, Rhode Island. Limnol Oceanogr 51: 1819–1831.

[67] Arai MN (1997) A functional biology of Scyphozoa. Chapman & Hall, New Your: 316 pp.

[68] Foshtomi MY, Abtahi B, Sari AE, Taheri M (2007) Ion composition and osmolarity of Caspian Sea ctenophore, *Mnemiopsis leidyi*, in different salinities. J Exp Mar Biol Ecol 352: 28–34.

[69] Mills CE (1984) Density is altered in hydromedusae and ctenophores in response to changes in salinity. Biol Bull 166: 206–215.

[70] Kideys AE, Romanova Z (2001) Distribution of gelatinous macrozooplankton in the southern Black Sea during 1996–1999. Mar Biol 139: 535–547.

[71] Purcell JE, Nemazie DA, Dorsey SE, Houde ED, Camble JC (1994) Predation mortality of bay anchovy *Anchoa mitchilli* eggs and larvae due to scyphomedusae and ctenophores in Chesapeake Bay. Mar Ecol Prog Ser 114: 47–58.

[72] Hays GC, Bastian T, Doyle TK, Fossette S, Gleiss AC, et al (2012) High activity and Lévy searches: jellyfish can search the water columan like fish. Proc R Soc B 279: 465–473. 10.1098/rspb.2011.0978 PMC3234559 21752825

